# Effect of combined colloid preloading and crystalloid coloading versus combined colloid and crystalloid coloading on maternal cardiac output during spinal anesthesia for cesarean section under combined prophylactic noradrenaline infusion

**DOI:** 10.3389/fmed.2025.1421947

**Published:** 2025-07-09

**Authors:** Xiang Gao, Yu Huang, Sumei Hu, Chuantao Lin, Yi You, Shihong Huang, Ming Liu, Jianying Yan

**Affiliations:** ^1^Department of Anesthesiology, Fujian Maternity and Child Health Hospital, Fuzhou, China; ^2^Department of Obstetrics, Fujian Maternity and Child Health Hospital, Fuzhou, China

**Keywords:** spinal anesthesia, cesarean section, cardiac output, noradrenaline, hypotension

## Abstract

**Objective:**

Hypotension is a common complication of spinal anesthesia during cesarean section, and no single intervention has been shown to eliminate maternal hypotension. Fluid loading strategies combined with vasopressor drug regimens can achieve this goal by maximizing cardiac output (CO) and minimizing the fall in systemic vascular resistance (SVR). However, the optimal fluid volume, type, and timing of administration have not been fully elucidated. Therefore, this study aimed to use Vigileo techniques in order to compare the effects of different fluid loading strategies on CO fluctuation under a norepinephrine infusion.

**Methods:**

We recruited 102 healthy term parturients scheduled for elective cesarean section under spinal anesthesia for this randomized double-blind study and divided them into two groups: the colloid preload followed by crystalloid coload group (500 ml each; Group 1, *n* = 51), and the colloid and crystalloid coload group (500 ml each; Group 2, *n* = 51). The infusion of norepinephrine was started after intrathecal injection. Vigileo was used to monitor invasive hemodynamic indices. Our primary outcome was standardized maternal cardiac output (CO) readings taken from spinal anesthesia until delivery. The secondary outcome measures were stroke volume (SV), systolic blood pressure (SBP), heart rate (HR), number of episodes of hypotension, hypertension, bradycardia, nausea/vomiting and total norepinephrine dose. Neonatal outcome was assessed by recording Apgar scores 1 and 5 min after delivery and by measuring umbilical arterial (UA) blood gases. Continuous hemodynamic monitoring was performed during the first 15 min after spinal anesthesia.

**Results:**

Baseline CO, SV, and SBP were similar for both groups. Before spinal anesthesia, CO (6.84 ± 1.18 vs. 5.51 ± 0.96 L/min, *P* < 0.001) was significantly higher in group 1 than group 2, but this increase was not sustained after spinal anesthesia (*P* > 0.05). SV (75.98 ± 13.01 vs. 66.37 ± 12.42 mL, *P* < 0.001) and SBP (124.84 ± 11.61 vs. 116.57 ± 7.57 mmHg, *P* < 0.001); followed a similar trend in the study. Only the largest percentage change in maternal HR (4.89 ± 11.89 vs. 10.38 ± 14.07, *P* = 0.036) was significantly different between the two groups. There were no significant differences between the two groups in terms of the maximum CO, SV, SBP, or HR after spinal anesthesia (*P* > 0.05). The maternal side effects and neonatal outcomes, were similar in two groups (*P* > 0.05).

**Conclusion:**

In combination with prophylactic norepinephrine infusion, 500-mL colloid preloading and 500-mL crystalloid coloading can significantly increase CO before spinal anesthesia for cesarean sections and provide improved hemodynamic stability after spinal anesthesia, with no difference in maternal or neonatal outcomes as compared to colloid and crystalloid coloading.

**Clinical trial registration:**

https://www.chictr.org.cn, identifier ChiCTR2300073333.

## Introduction

During cesarean section, spinal anesthesia results in extensive sympathetic nerve blockade to cause peripheral vasodilatation, hypotension, and consequently a reduction in cardiac output (CO) and placental blood flow, which is associated with negative maternal and fetal outcomes (1). To maintain haemodynamic stability after spinal anesthesia, the use of vasopressors is recommended to be supplemented with non-pharmacological measures and intravenous fluids (2). “Owing to its mild beta-adrenergic activity, norepinephrine is associated with improved heart rate (HR) and CO which has recently been described as a promising alternative to phenylephrine” (3, 4).

Fluid infused before or at the time of induction of spinal anesthesia is referred to as “preloading” and “coloading,” respectively. Crystalloid preloading has been shown to be ineffective on reducing the incidence and severity of hypotension, whereas colloids (e.g., hetastarch) are more effective in preventing hypotension and maintaining maternal CO (5–7). Carvalho et al. found that hetastarch preloading and coloading are equally effective in preventing hypotension after cesarean section spinal anesthesia, but the incidence of hypotension is still greater than 30% (8). JP Fitzgerald (9) found that colloid preloading was more effective than colloid coloading in preventing hypotension induced by spinal anesthesia for cesarean section after evaluating 109 studies. Other studies have shown that maternal CO has a greater effect on uteroplacental perfusion than blood pressure itself (10). Robson et al. (11) demonstrated a correlation between CO reduction and fetal acidosis during cesarean section under spinal anesthesia, but not between maternal hypotension and fetal acidosis. Arterial pressure measurements may not detect a low uterine placental perfusion. Recent international consensus guidelines recommend that clinicians pay more attention to arterial pressure and CO (2). Therefore, international consensus guidelines recommend clinical attention to CO rather than arterial blood pressure alone (2).

An increasing number of studies have focused on the effects of vasoactive drugs on the hemodynamics of spinal anesthesia for cesarean sections. However, few studies have evaluated the effects of different fluid loading methods combined with vasoactive drugs on hemodynamics. Therefore, we designed a double-blind, randomized controlled study to test the hypothesis that the combination of 500 mL colloid preloading and 500 mL crystalloid coloading with noradrenaline continuous infusion would have the same effect on CO variable as 500 ml colloid and 500 ml crystalloid coloaded with noradrenaline continuous infusion.

## Materials and experiments

This study adhered to the ethical principles outlined in the Declaration of Helsinki and was performed in accordance with the Consolidated Standards of Reporting Trials (CONSORT) guidelines (12). Ethical approval for this study (Ethical registration number: 2023 KY023) was provided by the Ethical Committee of Fujian Maternity and Child Health Hospital China (Chairperson Prof Pengming Sun) on 27 April 2023. All the participants provided written informed consent. The study was conducted from 7 July 2023, to 30 October 2023.

The inclusion criterion was single-born parturients aged 18–45 years with full-term pregnancies who were planned to undergo cesarean section at the Fujian Maternal and Child Health Hospital. The exclusion criteria were height < 150 cm, weight < 45 kg or > 100 kg, contraindication for spinal anesthesia (increased intracranial pressure, coagulation dysfunction, or local skin infection), chronic or pregnancy-induced hypertension, baseline systolic blood pressure (SBP) > 140 mmHg, hemoglobin < 10 g/dL, diabetes, cardiovascular, cerebrovascular, or kidney disease, hyperamniotic fluid or known fetal malformations, and multiple births.

The parturients did not receive any premedication. Upon arrival at the operating theater, patients were placed in a supine position with left uterine displacement, and standard non-invasive monitoring was applied, including non-invasive BP, pulse oximetry, and electrocardiography. An 18-gauge intravenous cannula was inserted into the left-hand vein, and a three-way stopcock was attached to the same line used for the infusion of intravenous fluid. A 20-gauge arterial line was inserted into the right radial artery after local anesthesia using an anesthetic skin patch. The arterial blood pressure was connected, and the transducer was placed at the level of the heart and zeroed. A Vigileo monitor (Edwards, EV1000A) was connected for continuous hemodynamic monitoring. After a brief settling period, baseline values for CO, stroke volume (SV), systolic BP (SBP), and maternal HR were obtained as the mean of three consecutive readings obtained at 3 min intervals, with a difference of less than 10%. No intravenous fluid preloading was administered.

Based on the random numbers generated by the computer, the puerperae were randomly divided into two groups at a 1:1 ratio: the colloid preloading and crystalloid coloading group (500 ml each; Group 1, *n* = 51) and the colloid and crystalloid coloading group (500 ml each; Group 2, *n* = 51). Random numbers and group information were placed in opaque envelopes that were opened according to the order in which the puerperae were included. The puerperae were grouped according to the envelope information and were not informed of the grouping results. All parturients received pressurized infusion using a bag pressurized to 250 mmHg. Group 1 received 6% w/v hydroxyethyl (HES) starch solution before induction of spinal anesthesia and 500 mL of Ringer’s lactate (RL) starting immediately after intrathecal injection. Patients in Group 2 received 500 mL of HES and 500 ml of RL, starting immediately after intrathecal injection. All study solutions in both groups were administered using a pressurizer adjusted to 250 mmHg. The fluid infusion was completed within 10 min in all subjects. No other fluids were administered to either group, except those used to maintain venous patency.

A 25-gauge pencil-point spinal needle was inserted in the midline at the estimated L3–L4 vertebral interspace with the patient in the left lateral position. After confirming the presence of CSF, 1.5 ml of 1% ropivacaine mixed with 1.0 ml of 10% dextrose and 0.5 ml of normal saline were injected intrathecally at a rate of 1 ml per 10 s. Immediately after completion of the intrathecal injection, the patients were returned to the supine position with left uterine displacement, and the infusion of norepinephrine was started at 0.05 μg/kg/min with an injection pump (MindRay BeneFunion nDSex), stopping norepinephrine pumping at the end of the surgery. The ice cube stimulation test was used for the sensory nerve block. The degree of sensory block was evaluated every 2 min after spinal anesthesia, and surgery was allowed when T6 was reached.

Three anesthesiologists participated in the study. The first anesthesiologist opened the sealed randomization envelope and placed the assigned study fluid in a simple pressurized infusion system. The anesthesiologist was solely responsible for the subsequent administration of fluid according to the study protocol. The second anesthesiologist administered the liquid and vasoactive drugs by diluting 0.4 mg of saline containing norepinephrine to 40 ml (50 ml syringe) with a connecting extension tube connected to tee at the end of intravenous indwelling needle to prepare the rescue drug norepinephrine (10 μg/ml). Bags of IV fluids and the proximal part of the IV tubing were covered with opaque cloth and shielded from the assessor’s view. A third anesthesiologist was responsible for spinal anesthesia. Throughout the procedure, the hemodynamic variables displayed by Vigileo’s monitors were obscured by opaque cardboard taped to the screen; thus, the anesthesia team had no way of knowing the CO or SV values.

During anesthesia and surgery, all parturients breathed air spontaneously, and additional oxygen was administered only when the pulse oximeter was lower than 95%. Hypotension was defined as a decrease in SBP of ≥ 20% below the baseline value. Hypotension was managed with a 5 μg intravenous bolus of norepinephrine. When the SBP decreased by 30% or more from baseline, a single bolus of 10 μg of norepinephrine was administered. If hypotension persisted, treatment with emergency pressors was repeated every 1 min. Reactive hypertension was defined as an increase in SBP to 120% of the baseline value. Hypertensive episodes were treated by stopping norepinephrine infusion, which was restarted when the SBP returned to < 120% of the baseline value. Bradycardia was defined as an HR < 50 beats/min. Atropine (0.5 mg) was injected intravenously when the HR was < 50 beats/min, and adverse reactions, such as nausea and vomiting, were observed. Tropisetron 5 mg was administered after the cause of hypotension was ruled out.

The primary outcome was maternal CO during the first 15 min after spinal anesthesia. Secondary outcome measures were stroke volume (SV), systolic blood pressure (SBP), heart rate (HR), number of episodes of hypotension, hypertension, bradycardia, nausea/vomiting, and total norepinephrine dose. Neonatal outcome was assessed by recording Apgar scores at 1 and 5 min after delivery and by measuring umbilical arterial (UA) blood gases.

### Statistical methods

Sample size calculation: In the pilot study, after colloid preloading, the preliminary experiment showed that the basic CO concentration in Group 1 was 7.11 ± 1.09 L/min after colloid coloading, whereas that in Group 2 was 5.80 ± 0.90 L/min. We assumed that group 1 had an increase in CO of at least 10% at 3–8 min after spinal anesthesia (peak). In Group 2, the CO increased by at least 20% 3–8 min after spinal anesthesia (peak period); the type-one error rate was 0.05, and the type-two error rate was 0.1. Two-sample *t*-tests assuming unequal variances were also conducted (PASS15.0; NCSS, LLC, Kaysville, UT, USA). A total of 92 patients were included (46 patients per group). Allowing for a 30% dropout rate, we planned to recruit 120 participants.

The Kolmogorov—Smirnov test was used to determine whether the continuous variables fit a normal distribution, and the independent sample *t*-test was used to compare the groups that fit a normal distribution. The Mann—Whitney U test was used for comparisons between groups that did not conform to a normal distribution. The χ^2^ test was used to compare the categorical variables. One-way repeated-measures ANOVA was conducted for CO, SV, systolic blood pressure, and heart rate. Data were analyzed using IBM SPSS Statistics (version 22.0; IBM Corp., Armonk, NY, USA). Statistical significance was set at *P* < 0.05.

## Results

**FIGURE 1:**
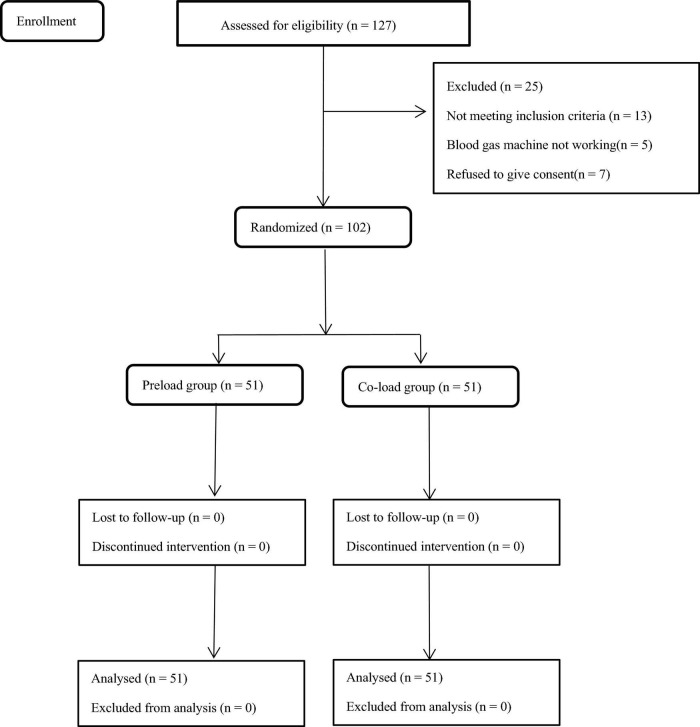
Recruitment flowchart.

A total of 127 parturients were recruited, of which 102 were enrolled in the study, 51 of whom were randomized in each group. The Consolidated Standards of Reporting Trials (CONSORT) recruitment diagram is shown in Figure 1.

Table 1 shows the parturients characteristics. Except for weight (65.64 ± 8.44 vs. 69.60 ± 8.29, *P* = 0.019) and BMI (25.67 ± 2.95 vs. 27.15 ± 2.89, *P* = 0.012) which showed significant differences in group comparisons, and the rest of the indices showed no significant differences in group comparisons (*P* > 0.05).

**TABLE 1:** Demographic and baseline characteristics.

	Group 1 (*n* = 51)	Group 2 (*n* = 51)	*P*-value
Age (years)	31.90 ± 4.51	30.41 ± 3.40	0.062
Weight (kg)	65.64 ± 8.44	69.60 ± 8.29	0.019
Height (cm)	159.84 ± 5.37	160.07 ± 5.52	0.835
BMI (kg/m^2^)	25.67 ± 2.95	27.15 ± 2.89	0.012
Gestational age (weeks)	38 [38, 39]	39 [38, 39]	0.224
Gravidity			0.552
Primigravida	26 (50.98)	22 (43.14)	
Multigravida	25 (49.02)	29 (56.86)	
Parity	0 [0, 1]	2 [1, 2]	0.716
Maximum sensory block	5 [5, 6]	5 [5, 6]	0.190
Spinal anesthesia–delivery time (min)	16.75 ± 4.48	15.67 ± 4.82	0.245

The values are presented as the mean ± SD or median (IQR).

The maximum hemodynamic changes in the parturient women (parturients) are shown in Table 2. Baseline CO, SV and, SBP were similar for both groups. Before spinal anesthesia, CO (6.84 ± 1.18 vs. 5.51 ± 0.96 L/min, *P* < 0.001) was significantly higher in in group 1 than group 2, but this increase was not sustained after spinal anesthesia (*P* > 0.05). SV (75.98 ± 13.01 vs. 66.37 ± 12.42 mL, *P* < 0.001) and SBP (124.84 ± 11.61 vs. 116.57 ± 7.57 mmHg, *P* < 0.001); followed a similar trend in the study. Only the largest percentage change in maternal HR (4.89 ± 11.89 vs. 10.38 ± 14.07, *P* = 0.036) was significantly different between the two groups. There were no significant differences between the two groups in terms of the maximum CO, SV, SBP, or HR after spinal anesthesia (*P* > 0.05).

**TABLE 2:** Changes in hemodynamic variables.

	Group 1 (*n* = 51)	Group 2 (*n* = 51)	*P*-value
**Baseline CO (L/min)**			
Baseline	5.50 ± 0.99	–	
After preload	6.84 ± 1.18	5.51 ± 0.96	< 0.001
Maximal change	7.60 ± 1.31	7.27 ± 1.27	0.203
% change	12.40 ± 16.70	33.92 ± 19.73	< 0.001
**Baseline SV (mL)**			
Baseline	69.41 ± 15.78	–	
After preload	75.98 ± 13.01	66.37 ± 12.42	< 0.001
Maximal change	87.16 ± 13.67	84.88 ± 17.17	0.461
% change	15.94 ± 14.76	29.09 ± 20.94	< 0.001
**Baseline SBP (mmHg)**			
Baseline	115.71 ± 6.68	–	
After preload	124.84 ± 11.61	116.57 ± 7.57	< 0.001
Maximal change	126.31 ± 9.86	124.10 ± 14.75	0.375
% change	1.58 ± 7.43	6.64 ± 11.98	0.012
**Baseline HR (beats/min)**			
Baseline	82.55 ± 8.19	–	
After preload	88.37 ± 10.39	85.00 ± 8.94	0.082
Maximal change	92.29 ± 12.34	93.76 ± 15.09	0.591
% change	4.89 ± 11.89	10.38 ± 14.07	0.036

The values are presented as the mean ± SD or median (IQR).

**FIGURE 2:**
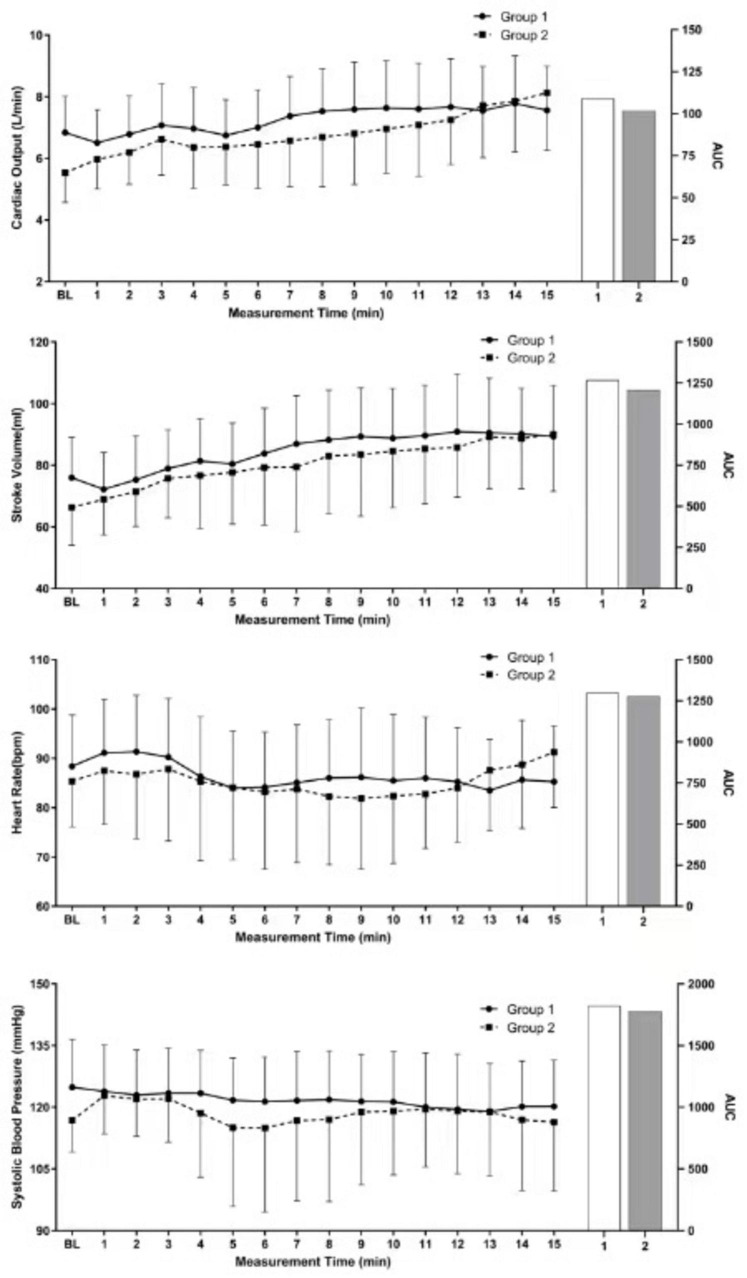
Repeated measures ANOVA results.

Repeated analysis of variance revealed significant difference between the two groups of parturients in terms of CO (*P* < 0.001), SV (*P* = 0.005), HR (*P* < 0.001), and SBP (*P* < 0.006) (Figure 2).

The incidence of hypotension, nausea, and vomiting was lower in Group 1 (5.88% vs. 29.41%, *P* = 0.003; 3.92% vs. 17.65%, *P* = 0.026); the frequency of norepinephrine intervention was lower (0 [0, 0] vs. 0 [0, 10], *P* < 0.001); and the incidence of hypertension and bradycardia, neonatal blood gas analysis, and Apgar score were not significantly different among the groups (*P* > 0.05) (Table 3).

**TABLE 3:** Maternal and neonatal outcomes.

	Group 1 (*n* = 51)	Group 2 (*n* = 51)	*P*-value
Hypotension–rescue bolus (%)	3 (5.88)	15 (29.41)	0.003
Hypertension (%)	2 (3.92)	4 (7.84)	0.678
Bradycardia (%)	0 (0.00)	0 (0.00)	> 0.999
Nausea or vomiting (%)	2 (3.92)	9 (17.65)	0.026
NE total dose (ug)	0 [0, 0]	0 [0, 10]	< 0.001
**Umbilical artery**			
pH	7.35 ± 0.04	7.35 ± 0.05	0.861
PCO2	46.16 ± 7.75	46.75 ± 9.17	0.781
PO2	21.08 ± 8.85	21.08 ± 11.27	0.998
Base excess	−1.03 ± 2.91	−0.37 ± 1.84	0.297
Lac	1.54 ± 0.30	1.71 ± 0.50	0.082
HCO3	25.36 ± 2.37	25.43 ± 2.69	0.912
**Apgar scores**			
1 min	10[10, 10]	10[10, 10]	0.080
5 min	10[10, 10]	10[10, 10]	1.0

The values are presented as the mean ± SD or median (IQR).

## Discussion

The results of this study showed that colloid fluid preloading significantly increased the CO, SV, and SBP of the puerperae; reduced the incidence of hypotension, nausea, and vomiting; and had no significant effect on neonatal prognosis. However, there was no significant difference in the maximum hemodynamic value after colloid preloading before spinal anesthesia combined with crystalloid coloading after anesthesia versus colloid coloading combined with crystalloid coloading after spinal anesthesia.

Due to the sympathetic blocking effect of spinal anesthesia, maternal blood pressure is often significantly reduced after spinal anesthesia owing to peripheral artery dilation and reduced vascular resistance, but the rise in SV resulting from the relief of caval compression together with an increase in venous return, CO increased from the preoperative state to a peak immediately following the delivery of the fetus and placenta (10). Because the uterus cannot regulate itself, uterine placental perfusion often depends on maternal CO (13); therefore, monitoring and maintenance of maternal CO are clinically important. In this study, the hemodynamic indices, including CO, SV, and SBP, significantly increased after a preloading of 500 ml of colloid solution before spinal anesthesia, and stability was better maintained after spinal anesthesia. This finding indicates that the administration of colloid solution preloading before spinal anesthesia has certain advantages. First, the purpose of preloading is to replenish the intravascular volume before the relatively effective circulation of blood volume becomes insufficient, owing to vasodilation induced by spinal anesthesia. In this study, a preloaded fluid volume of 500 ml did not significantly increase the circulating blood volume, which could lead to adverse events such as circulating overload and pulmonary edema. Second, the administration of colloid solutions reduces this physiological effect compared to the rapid distribution of the crystalloids, which causes 20% of the crystalloid to remain in circulation. Owing to the longer intravascular residence time, the dilatation effect is fully retained after spinal anesthesia, even when applied as a preloading (14, 15). Finally, the dilatation effect can be enhanced by colloid preloading during the peak period of vasodilation caused by spinal anesthesia.

Postanesthesia hypotension is believed to be due to decreased venous return, ventricular filling, and CO; therefore, volume management has been widely used as the primary coping strategy to prevent or treat post-anesthesia hypotension during cesarean section (16). With further research, it was found that the advantages of colloids are obviously superior those of crystalloid. Because colloids have larger molecules and cannot easily cross the endothelium, they stay in the vascular space longer, provide rapid plasma expansion, increase osmotic pressure, and improve microcirculatory organ perfusion (17). Park et al. (18) reported no significant differences in the incidence of hypotension among three groups after preloading with crystalloid solution at 10, 20, or 30 ml/kg, and 30 mL/kg preloading did not improve hemodynamics or reduce the demand for ephedrine. This dose also increases the risk of adverse events such as pulmonary edema. Tamilselvan et al. (19) compared the effects of 500 and 1,000 mL of hydroxyethyl starch preloading with 1,500 mL of crystalloid preloading on CO measured by Doppler imaging of the sternal bone. preloading increased CO, which remained above baseline only in patients receiving 1,000 mL of hydroxyethyl starch. Teoh and Sia (20) compared a preloading and a coloading of 15 ml/kg of 6% HES, and reported that the CO increased significantly after 5 min of preloading and then tended to stabilize. CO increased gradually after coloading, but some differences remained compared to those observed after preloading, and the effect of preloading was better. However, there were no significant differences in adverse maternal events or neonatal outcomes between the two groups. Similar results were obtained in this study. The hemodynamic indices, including CO, SV, and SBP, increased significantly after the colloid solution was prefilled, and the increase was within the acceptable range. Moreover, this approach has certain advantages over colloid liquid and crystalloid liquid co-loading within 15 min after spinal anesthesia. Therefore, the results of this study provide a practical basis for the further application of colloid fluid preloading and coloading in obstetric anesthesia.

Contraction of the peripheral vasculature and improvement of peripheral vascular resistance are key to coping with physiological changes in hypotension after spinal anesthesia. Therefore, vasoactive drugs, especially α-receptor agonists, are a key therapeutic strategy for the prevention and treatment of post-anesthesia hypotension (21). Norepinephrine (NE) is widely used to treat hypotension after spinal anesthesia. Because it stimulates α and β receptors simultaneously, norepinephrine improves maternal hemodynamic stability and reduces the incidence of bradycardia and is gradually being considered as an alternative to the guided recommended drug phenylephrine (22). Mohta et al. (23) compared 100 μg of phenylephrine and 5 μg of norepinephrine in the treatment of post-anesthesia hypotension (systolic blood pressure < 80% or absolute value < 100 mmHg); the incidence of sinus bradycardia was 37.8% and 22.2%, respectively (*P* = 0.167). There was no difference in the frequency of intervention for hypotension; however, the overall bolus intervention frequency was higher in the phenylephrine group (*P* = 0.01), and the pH value of the umbilical artery was higher (*P* = 0.034). Belin et al. (24) compared 0.5 μg/kg/min phenylephrine with 0.05 μg/kg/min norepinephrine to maintain maternal SBP above 90% of the baseline value and showed that the CI in the norepinephrine group was maintained at 90%–100% of the baseline value. However, the CI was maintained at slightly lower values in the phenylephrine group (81%–88% of baseline, *p* = 0.001). The percentage of elapsed time with a mean maternal BP < 65 mm Hg and with systolic BP < 80% of the baseline value was higher in the phenylephrine group. Hasanin et al. (25) studied the use of different infusion rates of noradrenaline to adjust the infusion rate under crystalloid coloading during spinal anesthesia and reported that the optimal dose of noradrenaline infusion for preventing spinal-induced hypotension was 0.05 μg/kg/min, which was consistent with the dose selected in this study, but the incidence of hypotension was 24.7%. It was similar to the colloid coloading preloading combined with crystalloid coloading group, which was much greater than that in the colloid preloading combined with crystalloid coloading group (5.8%). Preloading colloid may increase blood volume and rapid fluid redistribution into the interstitium. Therefore, this approach increases the CO value and reduces the incidence of hypotension. A new systematic review and network meta-analysis compared methods of preventing hypotension in parturients undergoing spinal anesthesia after cesarean section from the most effective to the least effective as follows: metaraminol, norepinephrine, phenylephrine, leg compression, ephedrine, colloid administered before induction of anesthesia, angiotensin, colloid given after induction of anesthesia, mephentermine, crystalloid administered after induction of anesthesia, and crystalloid administered before induction of anesthesia (26). Norepinephrine, whether administered as a bolus or infusion, have the potential to induce hypertension and reflex bradycardia, which may adversely impact maternal cardiac output (27). Reactive hypertension may be a contributing factor to hemorrhagic stroke, particularly in patients with preeclampsia. The high frequency of these adverse events implies that the current infusion concentration may not be appropriate. Hence, the prompt adjustment of prophylactic vasopressors dosage (discontinuation or reduction) is a crucial measure. Our findings contribute to a growing body of research verifying both the efficacy and safety of prophylactic norepinephrine infusion to prevent postspinal anesthesia hypotension during cesarean section.

This study had several limitations. First, central venous access was not available to determine the central venous pressure required to measure puerperal peripheral vascular resistance. Decreased peripheral vascular resistance is an important physiological change in patients under spinal anesthesia, and the determination of this index will help further clarify the advantages of the volume strategy and the application of vasoactive drugs. Second, we did not explore higher capacity management. In this study, a total liquid volume of 1,000 ml and colloid solution volume of only 500 ml were used. Different capacity management strategies may have affected these results. In the end, norepinephrine, being a major α1-agonist, leads to the increase of preload by promoting vasoconstriction, which in turn leads to increase of SV, CO, and SBP, Also, by its small effect on myocardial β1-receptors it has positive inotropic effect, which also contributes to the increase of CO by a small amount. So, (28) these norepinephrine actions present a bias effect on CO, SV, and SBP. Although norepinephrine infusion and boluses might influence CO, the same was given in the both groups. In addition, the number of norepinephrine boluses was recorded and it was higher in group 2, group 1 was more stabile, and that the protocol used in this group contributes to haemodynamic stability. Future studies may consider observing the effect of fluid loading on CO without norepinephrine infusion.

In conclusion, in patients undergoing cesarean section, colloid preloading combined with crystalloid coloading after anesthesia combined with noradrenaline infusion provided better hemodynamic stability and reduced maternal adverse events.

## Data Availability

The raw data supporting the conclusions of this article will be made available by the authors, without undue reservation.

## References

[1] Kranke P Geldner G Kienbaum P Gerbershagen H Chappell D Wallenborn J Treatment of spinal anaesthesia-induced hypotension with cafedrine/theodrenaline versus ephedrine during caesarean section: Results from HYPOTENS, a national, multicentre, prospective, noninterventional study. *Eur J Anaesthesiol.* (2021) 38:1067–76. 10.1097/EJA.0000000000001474 33625060 PMC8452326

[2] Kinsella S Carvalho B Dyer R Fernando R McDonnell N Mercier F International consensus statement on the management of hypotension with vasopressors during caesarean section under spinal anaesthesia. *Anaesthesia.* (2018) 73:71–92. 10.1111/anae.14080 29090733

[3] Heesen M Girard T Klimek M. Noradrenaline - at best it is not worse. A comparison with phenylephrine in women undergoing spinal anaesthesia for caesarean section. *Anaesthesia.* (2021) 76:743–7. 10.1111/anae.15363 33406274

[4] Theodoraki K Hadzilia S Valsamidis D Stamatakis E. Prevention of hypotension during elective cesarean section with a fixed-rate norepinephrine infusion versus a fixed-rate phenylephrine infusion. A double-blinded randomized controlled trial. *Int J Surg.* (2020) 84:41–9. 10.1016/j.ijsu.2020.10.006 33080415

[5] Gong R Liu X Li W Zhao J. Effects of colloid preload on the incidence of hypotension in spinal anesthesia for cesarean section: A systematic review and meta-analysis. *Chin Med J (Engl).* (2021) 134:1043–51. 10.1097/CM9.0000000000001477 33883404 PMC8116017

[6] Nishikawa K Yokoyama N Saito S Goto F. Comparison of effects of rapid colloid loading before and after spinal anesthesia on maternal hemodynamics and neonatal outcomes in cesarean section. *J Clin Monit Comput.* (2007) 21:125–9. 10.1007/s10877-006-9066-4 17265094

[7] Ko J Kim C Cho H Choi DH. A randomized trial of crystalloid versus colloid solution for prevention of hypotension during spinal or low-dose combined spinal-epidural anesthesia for elective cesarean delivery. *Int J Obstet Anesth.* (2007) 16:8–12. 10.1016/j.ijoa.2006.07.004 17125995

[8] Carvalho B Mercier F Riley E Brummel C Cohen S. Hetastarch co-loading is as effective as pre-loading for the prevention of hypotension following spinal anesthesia for cesarean delivery. *Int J Obstet Anesth.* (2009) 18:150–5. 10.1016/j.ijoa.2008.12.006 19223168

[9] Fitzgerald J Fedoruk K Jadin S Carvalho B Halpern S. Prevention of hypotension after spinal anaesthesia for caesarean section: A systematic review and network meta-analysis of randomised controlled trials. *Anaesthesia.* (2020) 75:109–21. 10.1111/anae.14841 31531852

[10] Ram M Lavie A Lev S Blecher Y Amikam U Shulman Y Cardiac hemodynamics before, during and after elective cesarean section under spinal anesthesia in low-risk women. *J Perinatol.* (2017) 37:793–9. 10.1038/jp.2017.53 28406485

[11] Robson S Boys R Rodeck C Morgan B. Maternal and fetal haemodynamic effects of spinal and extradural anaesthesia for elective caesarean section. *Br J Anaesth.* (1992) 68:54–9. 10.1093/bja/68.1.54 1739568

[12] Schulz K Altman D Moher D. CONSORT 2010 statement: Updated guidelines for reporting parallel group randomized trials. *Ann Intern Med.* (2010) 152:726–32. 10.7326/0003-4819-152-11-201006010-00232 20335313

[13] Lim G Facco F Nathan N Waters J Wong C Eltzschig HK. A review of the impact of obstetric anesthesia on maternal and neonatal outcomes. *Anesthesiology.* (2018) 129:192–215. 10.1097/ALN.0000000000002182 29561267 PMC6008182

[14] Theodoraki K Hadzilia S Valsamidis D Kalopita K Stamatakis E. Reply to Akça, B.; Bilotta, F. time and type of administered fluids during cesarean section might not matter for hemodynamic outcomes, but there are significant patient safety concerns regarding colloid use in parturients. Comment on;Theodoraki et al. colloid preload versus crystalloid co-load in the setting of norepinephrine infusion during cesarean section: Time and type of administered fluids do not matter. *J Clin Med*. (2023) 12:4754. 10.3390/jcm12144754 37510868 PMC10381518

[15] Theodoraki K Hadzilia S Valsamidis D Kalopita K Stamatakis E. Colloid preload versus crystalloid co-load in the setting of norepinephrine infusion during cesarean section: Time and type of administered fluids do not matter. *J Clin Med.* (2023) 12:1333. 10.3390/jcm12041333 36835869 PMC9964611

[16] McDonald S Fernando R Ashpole K Columb M. Maternal cardiac output changes after crystalloid or colloid coload following spinal anesthesia for elective cesarean delivery: A randomized controlled trial. *Anesth Analg.* (2011) 113:803–10. 10.1213/ANE.0b013e31822c0f08 21890886

[17] Guo L Xiong X Qin R Li Z Shi Y Xue W Prophylactic norepinephrine combined with 6% hydroxyethyl starch (130/0.4) co-load infusion for preventing postspinal anesthesia hypotension during cesarean section: A randomized, controlled, dose-finding trial. *Daru.* (2024) 32:1–9. 10.1007/s40199-023-00479-7 37812381 PMC11087382

[18] Park G Hauch M Curlin F Datta S Bader A. The effects of varying volumes of crystalloid administration before cesarean delivery on maternal hemodynamics and colloid osmotic pressure. *Anesth Analg.* (1996) 83:299–303. 10.1097/00000539-199608000-00017 8694309

[19] Tamilselvan P Fernando R Bray J Sodhi M Columb M. The effects of crystalloid and colloid preload on cardiac output in the parturient undergoing planned cesarean delivery under spinal anesthesia: A randomized trial. *Anesth Analg.* (2009) 109:1916–21. 10.1213/ANE.0b013e3181bbfdf6 19923521

[20] Teoh W Sia A. Colloid preload versus coload for spinal anesthesia for cesarean delivery: The effects on maternal cardiac output. *Anesth Analg.* (2009) 108:1592–8. 10.1213/ane.0b013e31819e016d 19372341

[21] Kuhn J Hauge T Rosseland L Dahl V Langesæter E. Hemodynamics of phenylephrine infusion versus lower extremity compression during spinal anesthesia for cesarean delivery: A randomized, double-blind, placebo-controlled study. *Anesth Analg.* (2016) 122:1120–9. 10.1213/ANE.0000000000001174 26991619

[22] Vallejo M Attaallah A Elzamzamy O Cifarelli D Phelps A Hobbs G An open-label randomized controlled clinical trial for comparison of continuous phenylephrine versus norepinephrine infusion in prevention of spinal hypotension during cesarean delivery. *Int J Obstet Anesth.* (2017) 29:18–25. 10.1016/j.ijoa.2016.08.005 27720613

[23] Mohta M Garg A Chilkoti G Malhotra RK. A randomised controlled trial of phenylephrine and noradrenaline boluses for treatment of postspinal hypotension during elective caesarean section. *Anaesthesia.* (2019) 74:850–5. 10.1111/anae.14675 31044424

[24] Belin O Casteres C Alouini S Le Pape M Dupont A Boulain T. Manually controlled, continuous infusion of phenylephrine or norepinephrine for maintenance of blood pressure and cardiac output during spinal anesthesia for cesarean delivery: A double-blinded randomized study. *Anesth Analg.* (2023) 136:540–50. 10.1213/ANE.0000000000006244 36279409

[25] Hasanin A Amin S Agiza N Elsayed M Refaat S Hussein H Norepinephrine infusion for preventing postspinal anesthesia hypotension during cesarean delivery: A randomized dose-finding trial. *Anesthesiology.* (2019) 130:55–62. 10.1097/ALN.0000000000002483 30335625

[26] Chooi C Cox J Lumb R Middleton P Chemali M Emmett R Techniques for preventing hypotension during spinal anaesthesia for caesarean section. *Cochrane Database Syst Rev.* (2020) 7:CD002251. 10.1002/14651858.CD002251.pub4 32619039 PMC7387232

[27] Mohta M Harisinghani P Sethi A Agarwal D. Effect of different phenylephrine bolus doses for treatment of hypotension during spinal anaesthesia in patients undergoing elective caesarean section. *Anaesth Intensive Care.* (2015) 43:74–80. 10.1177/0310057X1504300111 25579292

[28] Wang X Mao M Zhang S Wang Z Xu S Shen X. Bolus norepinephrine and phenylephrine for maternal hypotension during elective cesarean section with spinal anesthesia: A randomized, double-blinded study. *Chin Med J (Engl).* (2020) 133:509–16. 10.1097/CM9.0000000000000621 31996543 PMC7065858

